# Treatment of Esophageal Perforation with Primary Closure and Reinforcement Using TachoSil

**Published:** 2017

**Authors:** Abolghasem Daneshvar Kakhki, Seyed Reza Saghebi, Farahnaz Sadegh Bigee

**Affiliations:** Tracheal Diseases Research Center, NRITLD, Shahid Beheshti University of Medical Sciences, Tehran, Iran

**Keywords:** Esophageal perforation, Treatment, Closure

## Abstract

Two patients with iatrogenic esophageal perforation following rigid esophagoscopy for foreign body removal were successfully treated with primary repair and reinforcement using a collagen patch coated with human fibrinogen and thrombin (TachoSil, Nycomed, Austria, Vienna). The clinical implication of this report is that TachoSil can be used to bolster the repair site of esophageal perforation.

## INTRODUCTION

Esophageal perforation is a major life-threatening event with high morbidity and mortality rates (1,2). Although many treatment modalities depend on the cause and location of the perforation, the patient’s condition, the interval between the injury and the initiation of therapy, and the surgeon’s experience, the most optimal treatment remains controversial and continues to evolve (3,4). Among the different types of treatment options, primary repair is the most preferred method for an otherwise healthy esophagus and is usually accompanied by implementation of tissue grafts to bolster the repair site and reduce the possibility of leakage (1,4). Many tissues have been utilized for this purpose, but according to our knowledge, use of collagen patches coated with human fibrinogen and thrombin (TachoSil) to seal the repair area has not considered as a popular technique to date. In this report, we present two cases of esophageal perforation successfully treated with primary repair and reinforcement using TachoSil patches.

## CASE SUMMARIES

### Case 1

**Figure 1.**
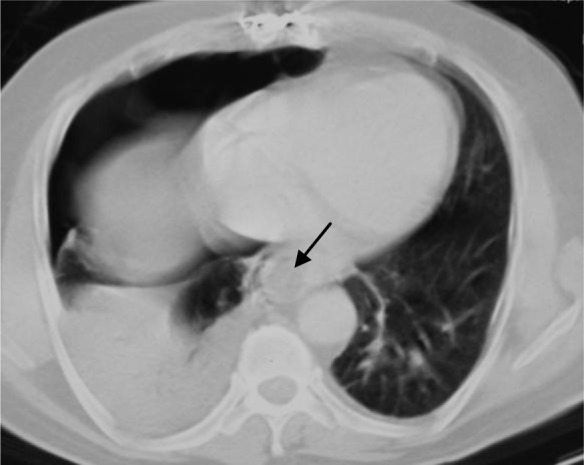
Chest CT demonstrates right side hydropneumothorax and impacted foreign body in the esophagus (arrow)

Owing to the inability to swallow and severe odynophagia after swallowing a large piece of meat, a 68-year-old man underwent flexible esophagoscopy, which revealed the swallowed meat stuck in the distal part of the esophagus. All attempts in another hospital to remove the foreign body by using rigid esophagoscopy were unsuccessful, and the patient was then referred to our center. He was ill and toxic on arrival, and rupture of the esophagus was subsequently confirmed based on computed tomography findings (Figure 1). The patient was prepared for urgent thoracotomy. Preoperative esophagoscopy revealed a full-thickness esophageal wall perforation approximately 37 cm from the incisor teeth just above the foreign body. The stuck swallowed meat was completely removed with grasping forceps, and reassessment of the esophagus did not show any abnormality. A right-sided posterolateral thoracotomy through the sixth intercostal space was performed and revealed a 5-cm longitudinal perforation in the distal part of the esophagus just above the esophageal hiatus and severe mediastinal inflammation around the perforation site. Despite the presence of signs of sepsis, including fever, tachypnea, tachycardia and loss of consciousness, and severe mediastinal inflammation, because of the relatively short interval between the onset of the primary injury and the operation, which was approximately 24 hours, a decision for primary repair was made. Vertical esophagomyotomy was performed to fully expose the damaged mucosa. After trimming the edges of the perforation, a secure two-layer interrupted closure was performed by using fine 4-0 Vicryl sutures. The repair site was buttressed by a 9.5- × 4.8-cm collagen patch coated with human fibrinogen and thrombin (TachoSil, Nycomed, Austria, Vienna).

**Figure 2.**
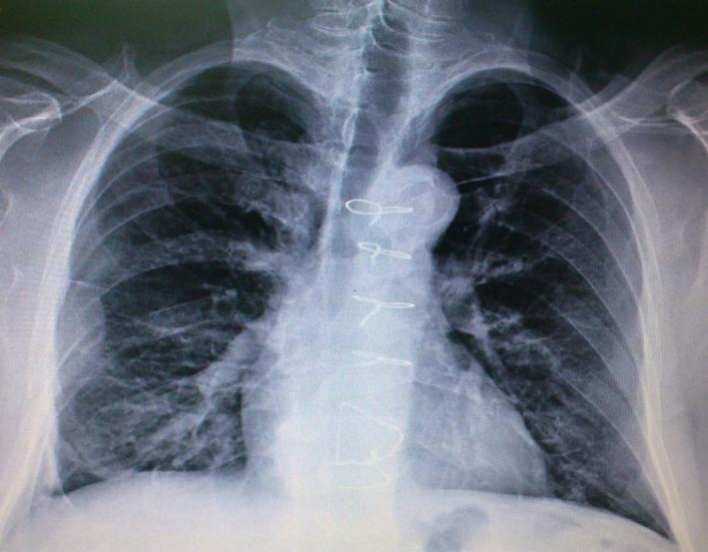
Chest X ray 2 months after the operation

The postoperative course was uneventful except for sputum retention, which was managed with flexible bronchoscopy on the third postoperative day. Gastrografin swallow radiographs obtained on postoperative day 9 did not demonstrate any leakage, and liquid diet was started and advanced gradually. The patient was discharged in a good condition on the 15th postoperative day. Owing to the history of esophageal foreign body in an elderly patient, he underwent flexible esophagoscopy and esophageal manometry 2 months later for detection of any undiagnosed esophageal disorder, but no abnormalities were found. The follow-up examination and chest radiography results were also normal (Figure 2).

### Case 2

**Figure 3.**
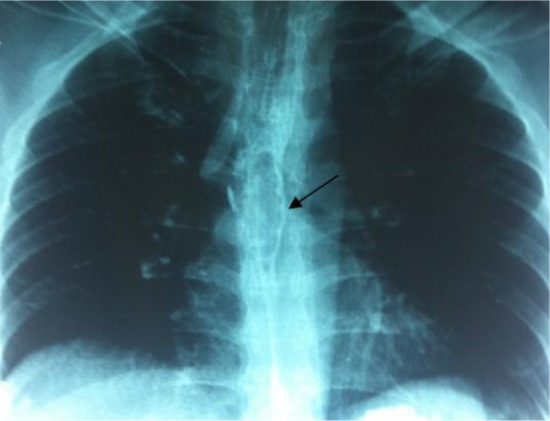
Chest X ray shows the presence of the foreign body in the middle part of the esophagus (arrow)

A 41-year-old man who presented with acute odynophagia and ***dysphagia*** after suddenly swallowing his dentures (Figure 3) was immediately referred to our center after unsuccessful attempts in another center to remove the foreign body by using rigid esophagoscopy. He was toxic upon arrival. Under the diagnosis of esophageal perforation, he was prepared for urgent thoracotomy. Preoperative esophagoscopy confirmed the presence of the foreign body in the middle part of the esophagus. However, the foreign body was stuck in the esophageal wall and we could not remove it. As in case 1, after esophagotomy at the site of the perforation and removal of the foreign body, primary repair was performed and a 9.5- × 4.8-cm TachoSil patch was used to buttress the repair site. On postoperative day 9, no leakage was seen on gastrografin swallow radiographs. He started to oral intake and was discharged on postoperative day 11 in a good condition.

## DISCUSSION

Esophageal perforation is a surgical emergency with high morbidity and mortality rates, and remains a therapeutic challenge despite improvement of clinical experience and innovative surgical techniques (1,2,4,5). An effective strategy for optimal treatment of esophageal perforation should include elimination of the source of infection and prevention of further contamination, extensive surgical drainage, broad-spectrum antibiotics, restoration of the integrity of the gastrointestinal tract, and adequate nutritional support (1,4, 6). Although the interval between the onset of perforation and treatment initiation is the most important prognostic factor, the cause and location of the perforation, the presence of concomitant esophageal disease, the severity of infection, and the surgeon’s experience are also important predictors of survival (1,4,5,7).

Management options for the treatment of esophageal perforation include primary repair with or without reinforcement, drainage alone, T-tube drainage, exclusion and diversion, esophageal resection with immediate or delayed reconstruction, endoluminal esophageal stent placement, fibrin glue application, endoscopic clipping, and sometimes, nonoperative management in selected patients (1,3–5,8).

Primary repair is the most preferred option and, in the absence of any underlying disease, is the surgical treatment of choice (1,4, 8). Early primary repair leads to a higher success rate, although some studies have shown that repair can be done after a delayed diagnosis (1,3,5,9,10). To prevent postoperative leakage, reinforcement of the repair site by using viable tissue grafts, including the pleura, intercostal muscles, rhomboid and latissimus dorsi muscles, omentum, diaphragm, pericardium or pericardial fat, gastric fundus and sternothyroid, and sternohyoid or sternoclidomastoid muscles, is recommended for cervical esophageal repair (1,4). Fibrin glue has also been used to primarily seal and reinforce a primary repair of an esophageal perforation, to improve adherence of a transposed flap, and to seal a layer of mesh placed over the repair site (11–13).

Collagen patches coated with fibrinogen and thrombin are currently available as commercial products to use for topical hemostasis or as sealant during surgery (14). TachoSil, which contains human fibrinogen, thrombin, and equine collagen, creates a fibrin clot at the surface of its placement through a process similar to the final steps of the natural blood clot formation (14). This product has been used to attain hemostasis and tissue sealing, and support sutures or occlude structures such as the bronchioles, lymph vessels, or bile ducts (14).

The clinical efficacy of TachoSil in many fields of abdominal surgery has been demonstrated by experimental and clinical studies (14–18). The usefulness of this product as a sealant has also been shown in thoracic surgery for the treatment of air leakage (19–23). Erdogan and colleagues used a fibrin tissue patch (TachoComb) to reinforce the repair site of esophageal perforation (24). Although the efficacy of using a fibrin tissue patch for reducing postoperative fistula was not statistically established, they concluded that surgical primary repair with reinforcement using a fibrin tissue patch is the most successful treatment option in the management of esophageal perforation.

In conclusion, based on several clinical or experimental studies that reported the efficacy of TachoSil as a useful sealant, we believe that using this patch instead of tissue grafts to reinforce the primary repair of an esophageal perforation may improve the seal of the repair and reduce operation time. However, because only a few studies have been conducted concerning the use of TachoSil to seal an esophageal perforation repair, further studies and follow-up are needed to reach any definitive conclusion in this regard.
